# Isometric Signal Processing under Information Geometric Framework

**DOI:** 10.3390/e21040332

**Published:** 2019-03-27

**Authors:** Hao Wu, Yongqiang Cheng, Hongqiang Wang

**Affiliations:** College of Electronic Science, National University of Defense Technology, Changsha 410073, China

**Keywords:** information geometry, intrinsic parameter submanifold, isometric signal processing

## Abstract

Information geometry is the study of the intrinsic geometric properties of manifolds consisting of a probability distribution and provides a deeper understanding of statistical inference. Based on this discipline, this letter reports on the influence of the signal processing on the geometric structure of the statistical manifold in terms of estimation issues. This letter defines the intrinsic parameter submanifold, which reflects the essential geometric characteristics of the estimation issues. Moreover, the intrinsic parameter submanifold is proven to be a tighter one after signal processing. In addition, the necessary and sufficient condition of invariant signal processing of the geometric structure, i.e., isometric signal processing, is given. Specifically, considering the processing with the linear form, the construction method of linear isometric signal processing is proposed, and its properties are presented in this letter.

## 1. Introduction

Information geometry was pioneered by Rao [1] in 1945, and the more concise framework was built up by Chentsov [2], Efron [3,4], and Amari [5]. In information geometry, the research object is the statistical manifold, which consists of a parameterized family of probability distributions with a topological structure, M={p(x;ξ)}. Given the Fisher information matrix as the Riemannian metric, the distance between any two points (probability distributions) can be calculated [6]. In such a manifold, the distance between two points stands for the intrinsic measure for the dissimilarity between two probability distributions [7]. As information geometry provides a new perspective on signal processing, there are many applications of it. In estimation issues, based on the Riemannian distance, the natural gradient has been employed [8,9,10]. The intrinsic Cramér–Rao bound is a tighter bound of both biased and unbiased estimators and derives from the Grassmann manifold [11]. In addition, the geometric structure (considering the distance between all pairs of points) can be used as an evaluation of the quality of the observation model, which has been applied in waveform optimization [12]. In optimization problems under the matrix constraint, the geometric structure was utilized [13,14,15]. Moreover, there are also many significant works of detection based on the distance [16,17,18,19,20]. Furthermore, in image processing, based on the Grassmann manifold, the target recognition in the SAR (Synthetic Aperture Radar) image is proposed [21].

As this new general theory has revealed the capability to solve statistical problems, the further development of information geometry demands the unambiguous relationship between the geometric structure and the intrinsic characteristic of common issues. This letter focuses on the influence of the signal processing on the statistical manifold in terms of estimation issues. In the estimation issues, the signal processing is the common means to mine for the information of a desired parameter. Accompanying signal processing, the geometric structure of the considered statistical manifold, to which the distribution of the observed data belongs, would change. The purpose of this letter is studying the geometric structure change accompanying signal processing and proposing an appropriate processing based on the change of the structure.

This research will be presented in the following way. At first, according to the essence of the estimation issues, the intrinsic parameter submanifold, which reflects the geometric characteristic of the issues, has been defined. Then, we show that the statistical manifold will become a tighter one after processing and give the necessary and sufficient condition of the invariant signal processing of the geometric structure (named isometric signal processing). Considering the more specific condition that the processing is linear, the construction method of linear isometric processing is proposed. Moreover, the properties of the constructed processing are presented.

The following notations are adopted in this paper: the math italic *x*, lowercase bold italic x, and uppercase bold A denote the scalars, vectors, and matrices, respectively. Constant matrix I indicates the identity matrix. Symbols ${\left(\cdot \right)}^{H}$, ${\left(\cdot \right)}^{T}$, and ${\left(\cdot \right)}^{*}$ indicate the conjugate transpose operator, transpose operator, and the complex conjugate, respectively. In addition, ${\left[A\right]}_{ij}$ indicates the *i*th row *j*th column element of matrix A, and rk(A) is the rank of matrix A. Moreover, A≥0 means that the matrix A is a positive semidefinite matrix. Finally, E(·) indicates the statistical expectation of a random variable.

## 2. Intrinsic Parameter Submanifold

Let M={p(x,ξ)} be a statistical manifold with coordinate system ξ, which consists of a family of probability distributions. Consider an estimation issue on the statistical manifold M; the observed data $x=(x_{1},x_{2},\cdots ,x_{N})$ belong to one of the probability distributions p(x,ξ) in M. Suppose the desired parameter θ is implied in parameter ξ and the relation between θ and ξ can be expressed as a mapping, h:θ↦ξ. As an instance, in the distance measurement of the pulse-Doppler radar, the desired distance *r* is embedded in the statistical mean μ of the observed data, i.e., μ=h(r)=P(t−2r/c) (P(t) means the pulse signal, and *c* is the velocity of light).

Actually, not all p(x,ξ) in M are concerned with the estimation issue; the considered probability distributions {p(x,h(θ))} not cover the whole manifold, they are only from a submanifold, which is the essential manifold in the issue. In the above example, the considered distributions are screened by the pulse signal P(t) (the statistical mean μ is able to be expressed as P(t−2r/c)).

**Definition 1** (Intrinsic parameter submanifold)**.** *The manifold S={p(x,h(θ))} is the intrinsic parameter submanifold of M={p(x,ξ)}, with coordinate system θ.*

**Figure 1:**
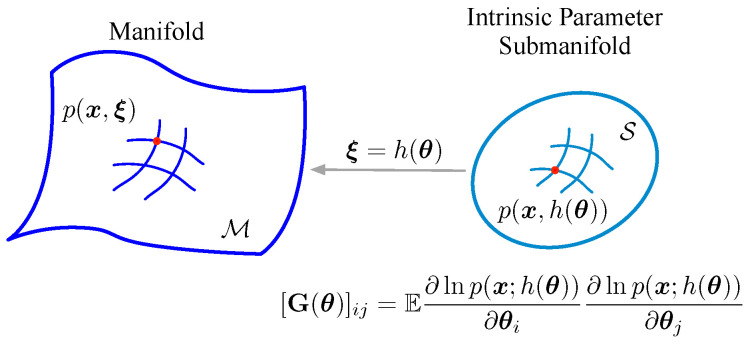
The intrinsic parameter submanifold.

The Riemannian metric of submanifold S is defined as $I_{x}\left(\theta \right)$, the Fisher information matrix associated with parameter θ, as in Figure 1. Actually, the distance of two points on the submanifold is defined by using the Riemannian metric [6].

**Remark** **1.** *When the Fisher information matrices $G_{1},G_{2}$ belonging to two observation models satisfy $G_{1}\geq G_{2}$, the observation model with $G_{1}$ is suggested to be better than another in terms of the estimation problem. The reason is that the distance $D_{1}\left(\theta_{1},\theta_{2}\right)$ (defined by $G_{1}$) is larger than $D_{2}\left(\theta_{1},\theta_{2}\right)$ (defined by $G_{2}$), because of the definition of the distance on the manifold. That means the two parameters $\theta_{1},\theta_{2}$ are easier to discriminate in the manifold with $G_{1}$ than $G_{2}$.*

Furthermore, the above remark also can be explained in traditional statistical signal processing. In estimation theory, the Fisher information also plays an important role, as the CRLB (Cramér–Rao Lower Bound) inequality. Therefore, in the traditional estimation theory, the same conclusion can be educed.

## 3. Signal Processing on the Intrinsic Parameter Submanifold

### 3.1. Geometric Structure Change by Signal Processing

In estimation issues, the signal is often processed to another form to obtain accurate estimates. Consider the signal processing y=g(x), where x indicates the original signal and y is the processed signal. The signal processing often accompanies the varying of the statistical manifold, specially the varying of the Riemannian metric.

One of the most vital factors of the submanifold in terms of estimation issues is its Riemannian metric, because the distance, representing the similarity, between two parameters is defined by it. Suppose the intrinsic parameter submanifold of *x* and *y* are S and $S^{\prime }$, respectively. The Riemannian metrics of S and $S^{\prime }$ are $G_{S}$ and $G_{S^{\prime }}$, respectively. If the PDFs (Probability Density Functions) $p_{x}\left(x;\theta \right)$, $p_{y}\left(y;\theta \right)$, and $p_{\mathrm{xy}}\left(x,y;\theta \right)$ obey the boundary condition [22], then the Fisher information satisfies the following equation [22,23],

(1) $$-E\frac{\partial^{2}\ln p_{x,y}\left(x,y;\theta \right)}{\partial \theta_{i}\partial \theta_{j}}=-E\frac{\partial^{2}\ln p_{x|y}\left(x|y;\theta \right)}{\partial \theta_{i}\partial \theta_{j}}-E\frac{\partial^{2}\ln p_{y}\left(y;\theta \right)}{\partial \theta_{i}\partial \theta_{j}},$$

(2) $$-E\frac{\partial^{2}\ln p_{x,y}\left(x,y;\theta \right)}{\partial \theta_{i}\partial \theta_{j}}\geq -E\frac{\partial^{2}\ln p_{y}\left(y;\theta \right)}{\partial \theta_{i}\partial \theta_{j}}.$$

Because y is produced by x via y=g(x), the following equation has been established.

(3) $$-E\frac{\partial^{2}\ln p_{x,y}\left(x,y;\theta \right)}{\partial \theta_{i}\partial \theta_{j}}=-E\frac{\partial^{2}\ln p_{x}\left(x;\theta \right)}{\partial \theta_{i}\partial \theta_{j}}\;\left(y=g\left(x\right)\right)$$

**Proof.**  Because $p_{xy}\left(x,y;\theta \right)=0$ for y≠g(x), then the $p_{x,y}\left(x,y;\theta \right)$ can be expressed as $p_{x,y}\left(x,y;\theta \right)=p_{x}\left(x;\theta \right)\delta \left(y-g\left(x\right)\right)$, the Fisher information can be simplified:

(4) $$-E\frac{\partial^{2}\ln p_{x,y}\left(x,y;\theta \right)}{\partial \theta_{i}\partial \theta_{j}}=-E\frac{\partial^{2}\ln p_{x}\left(x;\theta \right)}{\partial \theta_{i}\partial \theta_{j}}-E\frac{\partial^{2}\ln\delta (y-g(x))}{\partial \theta_{i}\partial \theta_{j}}=-E\frac{\partial^{2}\ln p_{x}\left(x;\theta \right)}{\partial \theta_{i}\partial \theta_{j}}.$$

 □

Then, the following lemma holds.

**Lemma** **1.** *The Riemannian metrics $G_{S}$ and $G_{S^{\prime }}$ satisfy, ∀θ:*

(5) $${\left[G_{S^{\prime }}\left(\theta \right)\right]}_{ij}-E\frac{\partial^{2}\ln p_{x|y}\left(x|y;\theta \right)}{\partial \theta_{i}\partial \theta_{j}}={\left[G_{S}\left(\theta \right)\right]}_{ij}.$$

**Proof.**  By Equations (1) and (3), and the definitions of $G_{S}$ and $G_{S^{\prime }}$, the lemma has been established. □

**Corollary** **1.** *For each θ, $G_{S}\left(\theta \right)-G_{S^{\prime }}\left(\theta \right)$ is a positive semidefinite matrix, i.e., $G_{S}\left(\theta \right)\geq G_{S^{\prime }}\left(\theta \right)$.*

**Proof.**  By Equation (2), Equation (3), and the definitions of $G_{S}$ and $G_{S^{\prime }}$, the corollary has been established. □

**Figure 2:**
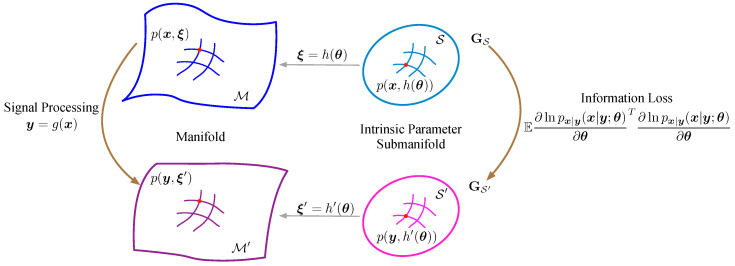
The signal processing on the intrinsic parameter submanifold.

Therefore, according to Lemma 1 and its corollary, the signal processing would result in Fisher information loss. As Figure 2 shows, the signal processing would turn the intrinsic parameter submanifold into a tighter one, i.e., discriminating two parameters turns out to be more difficult.

### 3.2. Isometric Signal Processing

As the above discussion, the appropriate signal processing should satisfy that the intrinsic parameter submanifold of processed signal is isometric to the original submanifold, i.e., the difference between any two parameters is unreduced.

**Definition 2** (Isometry)**.** *When $G_{S}\left(\theta \right)=G_{S^{\prime }}\left(\theta \right)$, the two intrinsic parameter submanifolds S and $S^{\prime }$ are isometric.*

Actually, the sufficient and necessary condition of the isometry of S and $S^{\prime }$ is as follows.

**Theorem** **1.** *If and only if y is the sufficient statistic of x, $G_{S}\left(\theta \right)=G_{S^{\prime }}\left(\theta \right)$.*

**Proof.**  For Lemma 1, the following relations are equivalent,

(6) $$G_{S}\left(\theta \right)=G_{S^{\prime }}\left(\theta \right)⟺E{\left(\frac{\partial \ln p_{x|y}\left(x|y;\theta \right)}{\partial \theta_{i}}\right)}^{2}\;=0\left(\forall \;i\right)⟺\frac{\partial p_{x|y}\left(x|y;\theta \right)}{\partial \theta_{i}}\overset{a.e.}{=}0\left(\forall \;i\right).$$

That means $p_{x|y}\left(x|y;\theta \right)$ is irrelevant to parameter θ, i.e., y is the sufficient statistic of x. □

The theorem suggests to use the test statistic to estimate the desired parameter, in the information geometry view. Actually, this conclusion also can be ensured in traditional estimation theory. For the Rao–Blackwell theorem [24], for any estimator $\hat{\theta }\left(x\right)$, the estimator $\overset{ˇ}{\theta }\left(y\right)=E\left(\hat{\theta }\left(x\right)|y\right)$ is the better estimator, i.e., $E{\left(\overset{ˇ}{\theta }\left(y\right)-\theta \right)}^{2}\leq E{\left(\hat{\theta }\left(x\right)-\theta \right)}^{2}$, when y=g(x) is the sufficient statistic. This theorem indicates that designing the estimator using the sufficient statistic y is more appropriate, because for each estimator $\hat{\theta }\left(x\right)$ using the original signal x as input, there exists the estimator $\overset{ˇ}{\theta }\left(y\right)=E\left(\hat{\theta }\left(x\right)|y\right)$ using the sufficient statistic y as the input that is better than $\hat{\theta }\left(x\right)$. Furthermore, for the Lehmann–Scheffé theorem [25,26], when the sufficient statistic y is complete, if the estimator $\overset{ˇ}{\theta }\left(y\right)$ is unbiased, i.e., $E(\overset{ˇ}{\theta }\left(y\right))=\theta$, the estimator $\overset{ˇ}{\theta }\left(y\right)$ is the minimum-variance unbiased estimator.

**Corollary** **2.** *If g(x) is a reversible function, $G_{S}\left(\theta \right)=G_{S^{\prime }}\left(\theta \right)$.*

**Proof.**  If g(x) is a reversible function, the PDF of x and y satisfy:

(7) $$p_{y}\left(y;\theta \right)\frac{dg(x)}{dx}=p_{x}\left(x;\theta \right).$$

According to the Fisher–Neyman factorization theorem [27], y is the sufficient statistic of x, so $G_{S}\left(\theta \right)=G_{S^{\prime }}\left(\theta \right)$. □

When the processed signal y=g(x) is the sufficient statistic of x, the signal processing g(x) is the isometric signal processing. Specifically, the reversible processing is definitely isometric processing, such as DFT (Discrete Fourier Transformation, because the inverse discrete Fourier transformation can recover the original signal, i.e., DFT is a reversible process). Moreover, this conclusion is also encountered in traditional estimation theory as the Rao–Blackwell theorem and Lehmann–Scheffé theorem.

## 4. Linear Form of Signal Processing

In real works, the noise is often Gaussian or asymptotically Gaussian, and the common signal processing is linear, such as DFT, matched filter, coherent integration, etc. This section will discuss the linear form of signal processing on the Gaussian statistical manifold.

### 4.1. Model Formulation

The information, as the desired parameter, is usually embedded in the signal, and the signal is often contaminated by noise, which can be described as x=s(θ)+w, where s(θ) is the uncontaminated signal waveform, w is the Gaussian noise, and x is the signal. The linear signal processing can be expressed as a matrix form, y=Hx.

### 4.2. Fisher Information Loss of Linear Signal Processing

Suppose the linear form of signal processing is formed as y=Hx; x is the *m* dimension, and y is the *n* dimension, then the matrix H is the n×m dimension. If rk(H)<n, there are n−rk(H) rows, which are the linear combination of the rest of the rk(H) rows. Therefore, the PDF of y only depends on the rk(H) corresponding elements, and the Fisher information loss is equivalent to the loss of the submatrix consisting of such rk(H) rows. Therefore, for a convenient statement, rk(H) is assumed to be *n*, i.e., matrix H is row full rank.

The Fisher information loss will be discussed under WGN (White Gaussian Noise), at first. Then, the Fisher information under CGN (Colored Gaussian Noise) will be presented based on the results under WGN.

#### 4.2.1. White Gaussian Noise

Suppose the noise is WGN and with power $\sigma^{2}$, then the signal also obeys normal distribution $x∼N(s\left(\theta \right),\sigma^{2}I)$. As the property of the normal distribution, the distribution of y is also the normal distribution, but with different parameter $N(Hs\left(\theta \right),\sigma^{2}HH^{H})$. Calculate the Fisher information of x and Hx; the loss of information is:

(8) $$G_{\Delta }\left(\theta \right)=G_{S}\left(\theta \right)-G_{S^{\prime }}\left(\theta \right)=\frac{1}{\sigma^{2}}{\frac{\partial s(\theta )}{\partial \theta }}^{H}\left(I-H^{H}{\left(HH^{H}\right)}^{-1}H\right)\frac{\partial s(\theta )}{\partial \theta }.$$

#### 4.2.2. Colored Gaussian Noise

Suppose the noise is CGN and with covariance matrix C. According to the property of the Hermite positive definite matrix, the covariance matrix can be expressed as $C=DD^{H}$, where D is a reversible matrix.

According to Theorem 1, perform the reversible transformation $x^{*}=D^{-1}x$; the Fisher information is invariant, i.e., $G_{S}\left(\theta \right)=G_{S^{\prime }}\left(\theta \right)$, and the noise in $x^{*}$ is WGN. Performing the linear processing HD to $x^{*}$, the result is:

(9) $$HDx^{*}=HDD^{-1}x=y,$$

and the information loss can be calculated by Equation (8). Therefore, the loss of information is:

(10) $$G_{\Delta }\left(\theta \right)={\frac{\partial s(\theta )}{\partial \theta }}^{H}\left(C^{-1}-H^{H}{\left(HCH^{H}\right)}^{-1}H\right)\frac{\partial s(\theta )}{\partial \theta }.$$

### 4.3. The Construction of the Isometric Linear Form of Signal Processing

In the previous section, the sufficient and necessary condition of isometric signal processing was that y=g(x) is the sufficient statistic of x. However, the sufficient statistic of x is often difficult to obtain, and the isometric processing should be constructed in another way. This part will introduce the construction method of linear isometric signal processing.

As regards the previous discussion, the signal under CGN can be transformed to the signal under WGN without information loss. Therefore, the signal under WGN is discussed in this part. As for the condition of CGN, the signal can be white at first, then the next steps are the same as the WGN condition.

The linear isometric processing can be obtained in the following way. Firstly, solve the equation:

(11) $$\forall \theta ,\;{\frac{\partial s(\theta )}{\partial \theta }}^{H}v=0\;\left(v\in R^{m}\right).$$

Suppose the solution space is $V=\mathrm{span}\{v_{1},\;v_{2},\;\cdots ,\;v_{l}\}$ with dimension *l* and the orthogonal complement of V is $V^{⊥}$ with dimension n=m−l. Then, the desired signal processing is formed as:

(12) $$H={\left[v_{1}^{\prime },\;v_{2}^{\prime },\;\cdots ,\;v_{n}^{\prime }\right]}^{H},$$

where $v_{1}^{\prime },\;v_{2}^{\prime },\;\cdots ,\;v_{n}^{\prime }$ is the bias of $V^{⊥}$.

**Proposition** **1.** *$H={\left[v_{1}^{\prime },\;v_{2}^{\prime },\;\cdots ,\;v_{n}^{\prime }\right]}^{H}$ is the isometric processing.*

**Proof.**  Let $Q=I-H^{H}{\left(HH^{H}\right)}^{-1}H$. Because the non-zero eigenvalue of $H^{H}{\left(HH^{H}\right)}^{-1}H$ is equivalent to that of $HH^{H}{\left(HH^{H}\right)}^{-1}=I$, the eigenvalue of $H^{H}{\left(HH^{H}\right)}^{-1}H$ is one (*n* multiplicity) and zero (m−n multiplicity). Therefore, the eigenvalue of Q is one (m−n multiplicity) and zero (*n* multiplicity). Then, as the matrix Q is the Hermitian symmetric matrix, it can be expressed as:

(13) $$Q=L\;\mathrm{diag}\left(1,\cdots ,1,0,\cdots ,0\right)L^{H}.$$

Consider the fact $QH^{H}=0$; the first *n* columns of HL must equal zero. That means the first *n* columns of L are the bias of V, and the rest of the columns are the bias of $V^{⊥}$, i.e.,

(14) $$L=[v_{1},\;\cdots ,v_{m-n},v_{1}^{\prime \prime },\;\cdots ,\;v_{n}^{\prime \prime }].$$

Because $v_{1},\;\cdots ,v_{m-n}$ is the solution of Equation (12),

(15) $$L^{H}\frac{\partial s(\theta )}{\partial \theta }={\left[v_{1},\;\cdots ,v_{m-n},v_{1}^{\prime \prime },\;\cdots ,\;v_{n}^{\prime \prime }\right]}^{H}\frac{\partial s(\theta )}{\partial \theta }={\left[0,\;\cdots ,0,v_{1}^{\prime \prime H}\frac{\partial s(\theta )}{\partial \theta },\;\cdots ,\;v_{n}^{\prime \prime H}\frac{\partial s(\theta )}{\partial \theta }\right]}^{T},$$

then:

(16) $${\frac{\partial s(\theta )}{\partial \theta }}^{H}L\;\mathrm{diag}\left(1,\cdots ,1,0,\cdots ,0\right)L^{H}\frac{\partial s(\theta )}{\partial \theta }=0,$$

i.e., the Fisher information loss is zero. □

According to the proposed construction method, the following proposition can be obtained.

**Proposition** **2.** *The matrix H is the isometric matrix with the minimal rows, i.e., the processed signal has the minimal length.*

**Proof.**  Let $H^{\prime }$ be the isometric matrix with dimension $n^{\prime }$ and $Q^{\prime }=I-H^{\prime H}{\left(H^{\prime }H^{\prime H}\right)}^{-1}H^{\prime }$. Similarly, the matrix also can be expressed as:

(17) $$Q^{\prime }=L^{\prime }\;\mathrm{diag}\left(1,\cdots ,1,0,\cdots ,0\right)L^{\prime H},$$

where the multiplicity of eigenvalue one is $m-n^{\prime }$.

As:

(18) $${\frac{\partial s(\theta )}{\partial \theta }}^{H}L^{\prime }\;\mathrm{diag}\left(1,\cdots ,1,0,\cdots ,0\right)L^{\prime H}\frac{\partial s(\theta )}{\partial \theta }=0,$$

the first $m-n^{\prime }$ rows of $L^{\prime H}\frac{\partial s(\theta )}{\partial \theta }$ must be zero, which means the first $m-n^{\prime }$ columns of L is the linear independent solution of Equation (12). However, the solution space $V=\mathrm{span}\{v_{1},\;v_{2},\;\cdots ,\;v_{l}\}$ has dimension m−n, so we can get $m-n^{\prime }\leq m-n$, i.e., $n^{\prime }\geq n$.

Therefore, the matrix H is the isometric matrix with the minimal rows. □

**Remark** **2.** *Because the first m−n columns of L are the linear independent solution of Equation (12), that means any element $v^{\prime }$ from $V^{⊥}$ satisfies that the first m−n elements of $L^{H}v^{\prime }$ equal zero. Therefore, the solution space of $Q^{\prime }x=0$ is $V^{⊥}$. Moreover, $H^{\prime }$ satisfies $Q^{\prime }H^{\prime H}=0$, so $H^{\prime }$ consists of the bias of $V^{⊥}$.*

In other words, the isometric matrix with dimension *n* is the equivalent matrix of H, which indicates that the proposed construction method can generate any isometric matrix with minimal rows.

#### Sample of the Construction

Consider the radar target detection scene: the radar emits the single frequency signal and receives the echo to obtain the distance and RCS (Radar-Cross-Section) information of the target. The observation model can be formulated as:

(19) $$x_{k}=A\exp\left(j2\pi f\left(kt_{\Delta }-\frac{2r}{c}\right)\right)+w_{k}\;k=1,\cdots ,N,$$

where *j* indicates the unit of the imaginary part, $t_{\Delta }$ is the sampling interval, *f* is the frequency of the emitted signal, *c* is the velocity of light, $w_{k}$ denotes WGN, *r* indicates the distance of the target, and *A* is the unknown amplitude, which contains the information of RCS. The desired parameter is θ=(A,r).

Firstly, the derivative is:

(20) $${\frac{\partial s(\theta )}{\partial \theta }}^{H}=\left[\begin{array}{ccc} \exp\left(j2\pi f\left(t_{\Delta }-\frac{2r}{c}\right)\right) & \cdots & \exp\left(j2\pi f\left(Nt_{\Delta }-\frac{2r}{c}\right)\right)\\ \frac{4jA\pi f}{c}\exp\left(j2\pi f\left(t_{\Delta }-\frac{2r}{c}\right)\right) & \cdots & \frac{4jA\pi f}{c}\exp\left(j2\pi f\left(Nt_{\Delta }-\frac{2r}{c}\right)\right) \end{array}\right].$$

Solve Equation (12); the orthogonal complement of the solution space is:

(21) $$\mathrm{span}\{\left(\exp\left(j2\pi ft_{\Delta }\right),\cdots ,\exp\left(j2\pi fNt_{\Delta }\right)\right)\}.$$

Therefore, the isometric processing is:

(22) $$y=\sum_{k=1}^{N}x_{k}\exp\left(j2\pi fkt_{\Delta }\right).$$

## 5. Conclusions

This letter focuses on the influence of signal processing on the geometric structure of the statistical manifold in estimation issues. Based on the intrinsic characteristics of the estimation issues, the intrinsic parameter submanifold is defined in this letter. Then, the intrinsic parameter submanifold is proven, which turns into a tighter one after signal processing. Moreover, we show that if and only if the processed signal is the sufficient statistic, the geometric structure of the intrinsic parameter submanifold is invariant. In addition, the construction method of the linear isometric signal processing is proposed. Moreover, the linear processing produced by the proposed method is shown with minimal rows (when it is represented as a matrix), i.e., the processed signal has the minimal length, and the proposed method can generate all linear isometry with minimal rows.
